# Differential Effects of Two Fermentable Carbohydrates on Central Appetite Regulation and Body Composition

**DOI:** 10.1371/journal.pone.0043263

**Published:** 2012-08-29

**Authors:** Tulika Arora, Ruey Leng Loo, Jelena Anastasovska, Glenn R. Gibson, Kieran M. Tuohy, Raj Kumar Sharma, Jonathan R. Swann, Eddie R. Deaville, Michele L. Sleeth, E. Louise Thomas, Elaine Holmes, Jimmy D. Bell, Gary Frost

**Affiliations:** 1 Nutrition and Dietetic Research Group, Department of Investigative Medicine, Imperial College London, Hammersmith Hospital, London, United Kingdom; 2 Metabolic and Molecular Imaging Group, MRC Clinical Sciences Centre, Imperial College London, Hammersmith Hospital, London, United Kingdom; 3 Department of Food and Nutritional Sciences, University of Reading, Reading, United Kingdom; 4 Department of Surgery & Cancer, Imperial College London, South Kensington Campus, London, United Kingdom; 5 Division of Animal Biochemistry, National Dairy Research Institute, Karnal, Haryana, India; 6 Nutrition and Nutrigenomics Group, Department of Food Quality and Nutrition, Research and Innovation Centre, Fondazione Edmund Mach di San Michele all'Adige, Trento, Italy; 7 Medway School of Pharmacy, Universities of Kent and Greenwich, Kent, United Kingdom; Paris Institute of Technology for Life, Food and Environmental Sciences, France

## Abstract

**Background:**

Obesity is rising at an alarming rate globally. Different fermentable carbohydrates have been shown to reduce obesity. The aim of the present study was to investigate if two different fermentable carbohydrates (inulin and β-glucan) exert similar effects on body composition and central appetite regulation in high fat fed mice.

**Methodology/Principal Findings:**

Thirty six C57BL/6 male mice were randomized and maintained for 8 weeks on a high fat diet containing 0% (w/w) fermentable carbohydrate, 10% (w/w) inulin or 10% (w/w) β-glucan individually. Fecal and cecal microbial changes were measured using fluorescent *in situ* hybridization, fecal metabolic profiling was obtained by proton nuclear magnetic resonance (^1^H NMR), colonic short chain fatty acids were measured by gas chromatography, body composition and hypothalamic neuronal activation were measured using magnetic resonance imaging (MRI) and manganese enhanced MRI (MEMRI), respectively, PYY (peptide YY) concentration was determined by radioimmunoassay, adipocyte cell size and number were also measured. Both inulin and β-glucan fed groups revealed significantly lower cumulative body weight gain compared with high fat controls. Energy intake was significantly lower in β-glucan than inulin fed mice, with the latter having the greatest effect on total adipose tissue content. Both groups also showed an increase in the numbers of *Bifidobacterium* and *Lactobacillus*-*Enterococcus* in cecal contents as well as feces. β- glucan appeared to have marked effects on suppressing MEMRI associated neuronal signals in the arcuate nucleus, ventromedial hypothalamus, paraventricular nucleus, periventricular nucleus and the nucleus of the tractus solitarius, suggesting a satiated state.

**Conclusions/Significance:**

Although both fermentable carbohydrates are protective against increased body weight gain, the lower body fat content induced by inulin may be metabolically advantageous. β-glucan appears to suppress neuronal activity in the hypothalamic appetite centers. Differential effects of fermentable carbohydrates open new possibilities for nutritionally targeting appetite regulation and body composition.

## Introduction

The gut microbiota is emerging as an important environmental factor associated with obesity and fat mass development [1]. Aberrant microflora profiles have been observed in both diet induced animal models of obesity [2] as well as in obese humans compared with lean controls [3]. Moreover, obese individuals on either a fat-restricted or carbohydrate-restricted low calorie diet have been shown to alter the gut bacteria compositions toward an increase of bacteroides and a reduction of firmicutes [3].

It is known that fermentable carbohydrates like inulin, oligofructose (fructans) and β-glucan undergo bacterial fermentation in the colon [4], [5]. Fermentable carbohydrates such as partially hydrolyzed guar gum and fructo-oligosaccharides have been shown to modulate the intestinal microbiota by increasing the proportions of bifidobacteria and lactobacilli in humans [6]. Supplementation of dietary fructans has also been shown to increase the level of anorectic gastrointestinal hormones such as glucagon like peptide-1 (GLP-1) and peptide YY (PYY) thereby reducing body weight gain in experimental animals [7]. The addition of oligofructose into the diet of obese adults for three weeks was shown to reduce body weight, suppress plasma ghrelin and enhance PYY levels [8]. Evidence shows that barley β-glucan also mediates a satiety effect through increased PYY and a reduction in ghrelin levels [9]. Moreover, the end products of bacterial fermentation, short chain fatty acids have been shown to regulate expression of the gut hormones implicated in satiety [10]. Previously we have shown that the increase in satiety as a result of resistant starch supplementation correlates with decrease in neuronal activation in the hypothalamic appetite centers using manganese enhanced magnetic resonance imaging (MEMRI) [11].

In the present study, we bring together a number of cutting-edge techniques to evaluate the efficacy of two different fermentable carbohydrates (β-glucan and oligofructose enriched inulin) in delaying the progression of obesity under high fat dietary conditions. We hypothesize that such carbohydrates may have a similar impact on body weight reduction but the underlying mechanisms by which they reduce weight gain may differ.

## Materials and Methods

### Animals and Treatments

**Figure 1:**
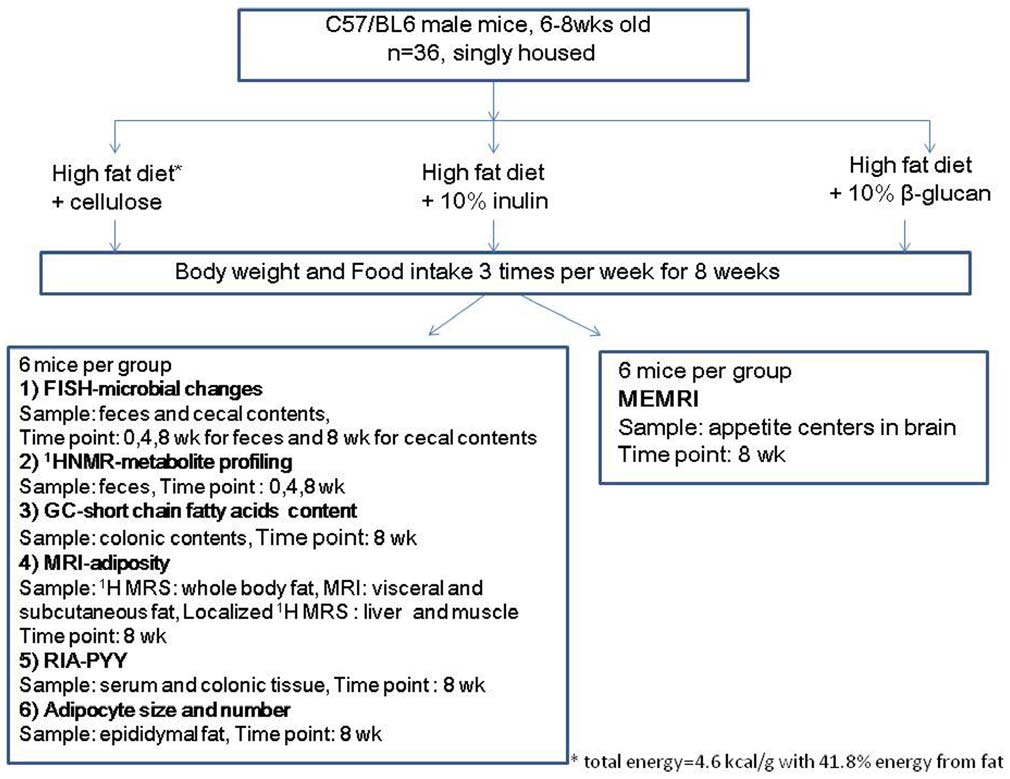
Flowchart showing the study design.

All animal procedures were performed in accordance with the UK Animals Scientific Procedures Act (1986). Thirty six male C57BL/6 mice (6–8 weeks old, Charles River, UK) were single housed under controlled temperature (21–23°C) and light conditions (12 h light-dark cycle; lights on at 07:00 h). Animals were randomized and assigned to three different groups (n = 12): High fat diet (HFD, with corn starch) as high fat control (HFD-C), HFD + oligofructose enriched inulin (Synergy™) (HFD-I) and HFD + β-glucan (Glucagel™) (HFD-BG). While Synergy™ is a fructan based preparation containing both long and short chain fructooligosaccharides, Glucagel™ is a highly rich (∼80%) barley derived β-glucan preparation (DKSH, London, UK). Both Synergy and Glucagel™ were mixed with the HFD individually in the ratio of 1∶9, the detailed composition of diets is given in the Table S1. The three diets were isocaloric, each contained the same total energy of 4.6 kcal/g, with 41.8% energy from fat. HFD diet was made isocaloric by the addition of cellulose. The diets were fed *ad libitum* for 8 weeks to respective group of animals. Body weights and food intake were measured three times per week. A schematic diagram outlining the study design is shown in Fig. 1.

### Fluorescent in situ hybridization (FISH)

The protocol was followed as previously described by Martin-Pelaez *et al*. [12] See method S1 for details. The probes used were Lab158 [13], Bif164 [14], Erec482 [15] and Mib663 [16] to enumerate *Lactobacillus-Enterococcus*, bifidobacteria, *Eubacterium rectale-Clostridium coccoides* and mouse intestinal bacteria belonging to *Bacteroides* subgroup in the phylum *Cytophaga-Flavobacter-Bacteroides*, respectively. Total cell counts were obtained with DAPI (4′,6-diamidino-2-phenylindole dihydrochloride) staining.

### ^1^H nuclear magnetic resonance (NMR) spectroscopy

Fecal samples were prepared and analyzed by ^1^H NMR using method adapted from Saric *et al.* [17]. See method S1 for details.

### Gas chromatography

Short chain fatty acids (SCFA) in the colonic contents were determined by gas chromatography. The method used was adapted from Richardson *et al* [18]. See method S1 for details.

### Magnetic resonance studies

A combination of magnetic resonance imaging and spectroscopy (MRI/S) techniques explained below were carried out to determine body composition in terms of total body fat content and distribution, and ectopic lipid levels in liver and muscle tissue. We also used MEMRI to assess the impact of fermentable carbohydrates on appetite centres in the hypothalamus.

**Whole body ^1^H MRS.** Whole body ^1^H MRS was performed to determine the whole body adiposity. The mice were fasted for 16 h and anesthetized with 1–2% isoflourane-oxygen mix which was maintained throughout the scan. The animals were scanned on a 4.7 Tesla Varian INOVA imaging system (Varian Inc, USA) using a pulse sequence with the following parameters: repetition time (TR) = 10 s, pulse angle = 45° and averages = 4. The spectra obtained were analyzed and body adiposity was calculated as previously described [11].

**Whole body MRI.** Whole body MRI was carried out to determine the amount of internal (epididymal and mesenteric fat) and total subcutaneous depots. Consecutive 2 mm thick slices were acquired using a spin-echo sequence with the following parameters: TR = 2.2 s, echo time (TE) = 20 ms, matrix size = 256×192, field of view (FOV) = 45×45 mm and averages = 2. The slices/images were then subjected to segmentation analysis (SliceOmatic™, Tomovision®, Canada) by an observer blinded to the experimental groups to obtain volumes and the masses of the different adipose tissue depots were calculated.

**Localized ^1^H MRS.** Localized ^1^H MRS was performed to assess the lipid content in the liver and muscle. The intrahepatocellular and intramyocellular lipid content was assessed by placing a voxel of 2×2×2 mm^3^ on a selected slice of the liver and muscle in the MR images. A PRESS sequence with the following parameters, TR = 10 s, TE = 9 ms and averages = 64, was applied on the voxel to obtain the spectra and relative percentage of lipid was determined by integration of the lipid peak [11].

**Manganese enhanced MRI (MEMRI).** MEMRI was performed to determine the neuronal activation in selected appetite centers of brain using 9.4 Tesla Varian INOVA imaging system (Varian Inc, USA) as described earlier. The mice for MEMRI were selected by the average body weight of each group and they had free access to food and water before the scan. Mice were anesthetized with 1–2% isoflourane-oxygen mix which was maintained throughout the scan. A fast spin-echo multi-slice sequence was applied with the following parameters: TR = 600 ms, TE = 10 ms, matrix size = 192×192, FOV = 25×25 mm and average = 1 acquiring 46×0.4 mm thick slices [19]. An array of 66 acquisitions was set up so that the 46 slices were acquired 66 times throughout the infusion. Maximum three scans were performed per day during the day light time and each scan lasted for 2 hr. Normalized percentage enhancement (NPE) in signal intensity was calculated in the hypothalamic appetite centers like arcuate nucleus (ARC), ventromedial hypothalamus (VMH), paraventricular nucleus (PVN), periventricular nucleus (PE), and the brainstem region like nucleus of tractus solitarius (NTS) [20].

### Adipocyte cell size and number

Adipocyte cell size and number were determined as reported earlier [21]–[23]. Briefly, white adipose tissue (epididymal depot) was collected and finely minced in Dulbecco's modified Eagle medium (DMEM) supplemented with 4% (w/v) Bovine serum albumin (BSA) and collagenase (1 mg/0.5 g tissue). The tissue suspension was incubated in a shaking water bath set at 140 cycles/min for 45 min at 37°C. After the tissue was completely digested, the suspension was filtered through a polypropylene mesh (400 µm) and washed twice using DMEM containing 4% (w/v) BSA and 1 mg/40 ml trypsin inhibitor. An aliquot (10 µl) of adipocytes was collected, diluted with trypan blue in 1∶1 ratio and a sample of the cells were counted in a haemocytometer. Images were taken of different sample regions of the grid on the haemocytometer for further analysis. Cell number and size (diameter, µm) was calculated from images using software CellProfiler (Masachusetts, USA) setting an appropriate threshold to exclude any cell debris.

### Colonic PYY extraction and radioimmunoassay

PYY was extracted from the colons by boiling for 15 minutes in 0.5 M acetic acid (10 ml/g wet weight of colon) as described previously [24]. Extracts were stored at −20°C until assayed. Colonic PYY and PYY_3–36_ immunoreactivity in serum was measured using radioimmunoassay [25].

### Statistical Analysis

All data was checked for normality, and results expressed as means ± standard errors (SE), unless otherwise stated. In the case of the gut microbiota, the data is presented as median and 95% confidence intervals. Unless otherwise stated, data was analyzed using two way ANOVA with post-hoc bonferroni correction. PYY and MRI results were analyzed by one way ANOVA using software GraphPad prism5. MEMRI timecourse data was analyzed using GEE with post-hoc Mann Whitney analysis [26].

For the acquired ^1^H NMR fecal spectra data, the spectra were initially referenced to sodium 3-trimethylsilyl [2,2,3,3-^2^H_4_] propionate, TSP (δ 0.0), corrected for baseline distortions and phased using an in-house routine written in Matlab® 7.3.0 (MathWorks, Natick, MA) and each spectrum was collected into the 0.005 ppm spectral region [27]. Analysis of the spectral data was performed initially using principal component analysis (PCA). Following PCA, pair wise orthogonal projection to latent structure discriminating analysis (OPLS-DA) models between different types of diet and at different time points were calculated separately using one predictive and one orthogonal component. Due to the small number of samples in this report, separation within the model was considered significant when p≤0.01. The computed regression coefficient plots [28] were used to identify the differential contribution of spectral regions in discrimination between class separations. Metabolites which were highly correlated to the class separations were assigned using published literature, statistical total correlation spectroscopy [29] and public databases.

## Results

### Body weight, food intake, tissue weights and adiposity data

**Figure 2:**
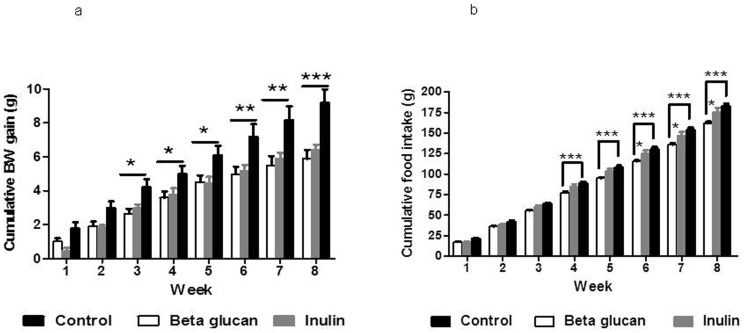
The effect of inulin and β-glucan supplementation over the 8-week dietary interventional period (a) weekly cumulative body weight gain, n = 12 per group (b) weekly cumulative food intake over the 8 week dietary intervention period, n = 12 per group. * = p<0.05, ** = p<0.01, *** = p<0.001. Key: HFD-C, high fat diet control; HFD-I, high fat diet+inulin; HFD-BG, high fat diet+β-glucan.

Both oligofructose enriched inulin (HFD-I) and β-glucan (HFD-BG) fed mice (n = 12 per group) displayed significantly lower (p<0.05) body weights at weeks 7 and 8 compared with high fat control mice (HFD-C). Cumulative body weight gain was significantly lower (p<0.05) in HFD-I and HFD-BG compared with HFD-C from week 3 onwards and this was maintained until the end of the dietary intervention giving a net reduction of 30% and 37%, respectively (Fig. 2a).

No difference in cumulative food intake was observed between HFD-C and HFD-I groups (Fig. 2b) despite a significantly lower cumulative body weight gain in the HFD-I group. The HFD-BG group showed a significantly lower cumulative food intake from week 4 as compared to HFD-C (p<0.001) and from week 6 as compared to HFD-I (p<0.05). Despite a significantly lower cumulative food intake in HFD-BG mice, no significant difference in cumulative body weight gain was observed between HFD-BG and HFD-I groups.

Epididymal fat mass was significantly lower (p<0.05) in HFD-I, while cecal weight (along with contents) was significantly higher (p<0.001) in both HFD-I and HFD-BG, compared with HFD-C (n = 6 per group, Table 1). The cecal weight of HFD-BG mice was significantly lower than that of HFD-I mice (p<0.01). Liver and colonic weights did not differ significantly.

**Table 1:** Effect of inulin and β-glucan supplementation on adiposity parameters and tissue weights in high fat fed mice (n = 6).

	HFD-C	HFD-I	HFD-BG
Epididymal adipose tissue (g)	1.14±0.16	0.59±0.10*	0.77±0.10
Whole body adiposity (%)	18.03±2.72	8.95±1.66*	12.17±1.92
Liver lipid content (%)	6.30±1.62	6.02±1.97	6.02±1.36
Muscle lipid content (%)	0.96±0.149	0.72±0.05	1.29±0.57
Internal fat (g)	2.17±0.46	1.23±0.17	1.49±0.27
Subcutaneous fat (g)	3.40±0.53	2.08±0.13	2.44±0.28
Adipocyte size ( µm)	122.25±10.2	72.95±8.72** ^,^ #	111.19±4.03
Adipocyte number (×10^7^)	1.43E+08	1.31E+08	1.86E+08
Liver size (g)	1.43±0.13	1.23±0.15	1.40±0.06
Caecum (with contents, g)	0.21±0.01	0.69±0.05*** ^,^ ##	0.49±0.03***
Caecum (without contents, g)	0.06±0.01	0.21±0.01*** ^,^ ##	0.14±0.01***
Colon (g)	0.13±0.01	0.19±0.02	0.14±0.02

Superscipt (*) shows the significant difference between HFD-I or HFD-BG vs. HFD-C.

* = P<0.05,

** = P<0.01,

*** = P<0.001.

Superscipt (#) shows the significant difference between HFD-I vs. HFD-BG.

# = P<0.05,

## = P<0.01.

### Body composition and adipocyte data

Whole body adiposity measured by ^1^H MRS (n = 6 per group) at week 8 showed a stepwise increase with HFD-I the lowest, followed by HFD-BG than HDF-C group. HFD-I (8.95±1.66%) was significantly lower (p<0.05) compared with both the HFD-BG (12.17±1.92%) and HDF-C (18.03±2.72%) groups (Table 1). There were no significant differences in internal and subcutaneous fat levels, however, a trend towards a lower internal and subcutaneous fat was observed in HFD-I and HFD-BG compared with HFD-C group. There were no significant differences observed in either liver or muscle lipid content between any of the three groups.

Adipocyte size was significantly lower (p<0.05) in the HFD-I (72.95±8.72 µm) compared to either the HFD-BG (111.19±4.03 µm) or HFD-C (122.25±10.2 µm), however, there was no significant change in adipocyte number between the different groups (Table 1).

### Gut microflora composition and SCFAs

**Figure 3:**
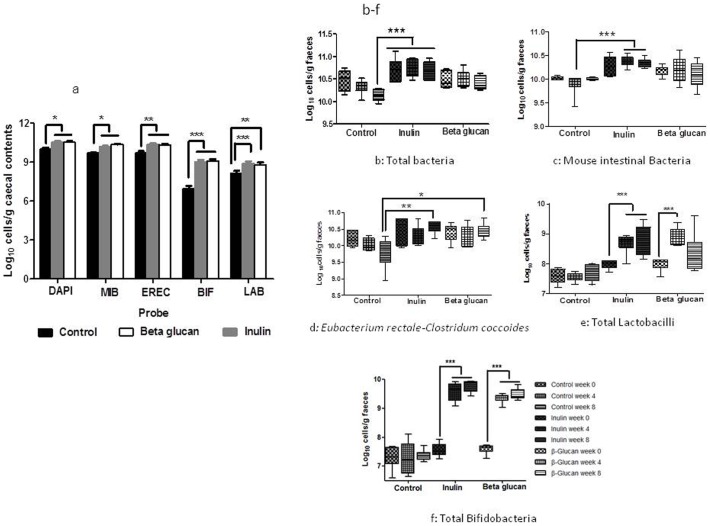
The effect of inulin and β-glucan supplementation on cecal and fecal microbial contents over the 8-week dietary interventional period (a) cecal microflora groups, n = 6 per group (b) fecal total bacteria microflora at week 0, 4 and 8, n = 6 per group: (c) fecal mouse intestinal bacteria, (d) fecal *Eubacterium rectal-Clostridium coccoides*; (e) fecal Lactobacilli; and (f) fecal Bifidobacteria. * = p<0.05, ** = p<0.01, *** = p<0.001. Key: HFD-C, high fat diet control; HFD-I, high fat diet+inulin; HFD-BG, high fat diet+β-glucan.

Fluorescence *in situ* hybridization (FISH) was carried out in both fecal pellets (collected at weeks 0, 4 and 8) and cecal contents at week 8 (when the mice were culled). A significant modulation of bacterial populations was observed in both the cecal and fecal contents (n = 6 per group, Fig. 3). Bacterial groups, *Lactobacillus-Enterococcus* and *Bifidobacterium* hybridized with probes Lab156 and Bif164, respectively, showed a significant increase in both HFD-I (p<0.001) and HFD-BG (p<0.01) compared to HFD-C in cecal contents post dietary intervention at week 8. HFD-I and HFD-BG fed mice also exhibited a significant increase in total bacterial (p<0.05), MIB (p<0.05) and EREC counts (p<0.01) compared to HFD-C (Fig. 3a).

Baseline fecal microbial contents were similar for mice fed with different diets (Fig. 3b–f). No significant changes in the fecal microbial contents were observed in HFD-C group over the 8 weeks dietary intervention. However, a non-significant reduction in the total bacteria and EREC was observed at week 8 in HFD-C group. The intake of fermentable carbohydrates resulted in a significant increase (p<0.001) in the numbers of total bifidobacteria and lactobacilli as compared to week 0, showing a similar pattern of microbial modulation as observed in cecal contents. Other bacterial groups, MIB bacteroides, residing within *Cytophaga-Flavobacter-Bacteroides* phylum (p<0.001) and EREC, *Eubacterium rectale-Clostridium coccoides* numbers (p<0.01) also increased in HFD-I as compared to HFD-C group.

Total level of SCFAs (acetate, propionate and butyrate) (n = 6 per group) were significantly higher in the HFD-BG group compared with the HFD-I group, which in turn was higher than the HFD-C group (HFD-BG: 34.4±2.5 mmol/mg; HFD-I: 27.8±2.1 mmol/mg; HFD-C: 17.7±4.1 mmol/mg, p<0.05) in the colonic contents. Acetate and propionate levels were significantly increased in HFD-BG and HFD-I compared with HFD-C (Acetate; HFD-BG: 30.2±2.4 mmol/mg; HFD-I: 25.1±3.1 mmol/mg; HFD-C: 16.7±3.1 mmol/mg, p<0.05: Propionate; HFD-BG: 3.0±1.2 mmol/mg; HFD-I: 2.0±0.9 mmol/mg; HFD-C: 0.6±0.3 mmol/mg, p<0.05). However, there was no significant difference observed in the butyrate levels among the three groups (HFD-BG: 1.1±0.3 mmol/mg; HFD-I: 0.7±0.2 mmol/mg; HFD-C: 0.4±0.1 mmol/mg, p = 0.260).

### MEMRI

**Figure 4:**
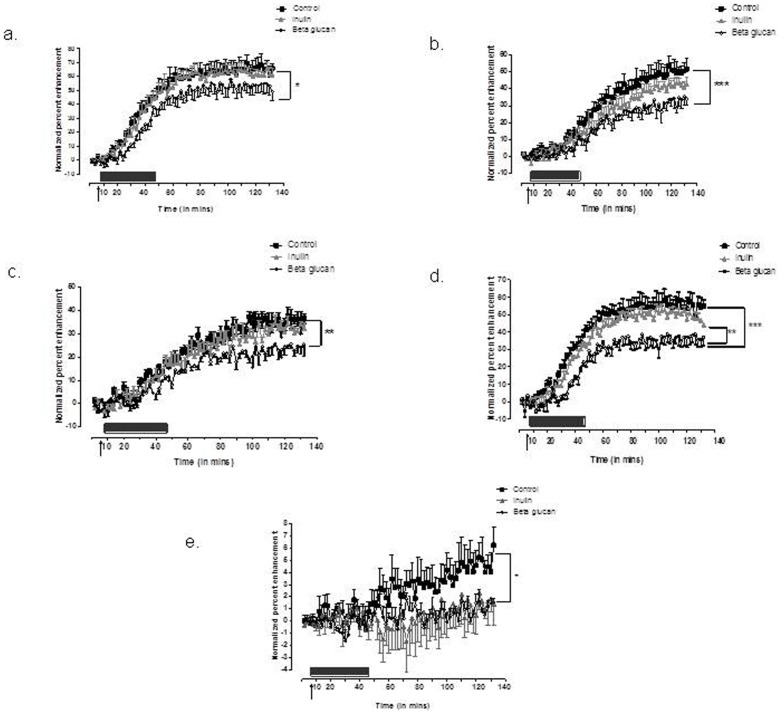
Representative baseline (pre-contrast) MRI images of the mouse brain showing assignment of regions of interest (ROIs) in various brain areas from which signal intensities (SI) were obtained. Time course of changes in SI (as a percentage of baseline) before and at various times after IV manganese chloride infusion in the (a) ARC, (b) VMH (c) PVN (d) PE (e) NTS. Data are presented as means of four consecutive image acquisitions±SEM. * = p<0.05, ** = p<0.01, *** = p<0.001 Key: ARC, arcuate nucleus; VMH, ventromedial hypothalamic nucleus; PVN, paraventricular hypothalamic nucleus; PE, periventricular nucleus; NTS, nucleus of solitaries tractus; HFD-C, high fat diet control; HFD-I, high fat diet+inulin; HFD-BG, high fat diet+β-glucan.

MEMRI was carried out at the end of the 8 weeks dietary intervention to measure neuronal activation in the appetite centers of brain (n = 6 per group) (Fig. 4a–e). Significantly lower signal intensities were observed in the ARC (p<0.05), VMH (p<0.001), PVN (p<0.01), PE (p<0.001) and NTS (p<0.05) in HFD-BG fed mice when compared to HFD-C group of mice. No significant difference was found between HFD-C and HFD-I groups for ARC, VMH, PVN and PE except the MEMRI pattern in NTS which was significantly (p<0.05) lower in HFD-I fed mice. HFD-I group generally showed higher signal intensities for the neuronal activation in the appetite centers of the brain when compared to the HFD-BG group. However, these changes were not significant except for the MEMRI pattern in PE (p<0.01) (Fig. 4d).

### Serum and colonic PYY content

There was no statistical difference observed in the serum (HFG-BG: 0.13±0.016 pmol/mL; HFD-I: 0.09±0.008 pmol/mL; and HFD-C: 0.10±0.012 pmol/mL) and colonic tissue (HFG-BG: 19.9±1.6 pmol/mL; HFD-I: 22.8±5.3 pmol/mL; and HFD-C: 27.3±3.7 pmol/mL) concentrations of PYY.

### NMR results

Fecal pellets collected at week 0, 4 and 8 were measured by ^1^H NMR spectroscopy to obtain the spectral profiles and to analyze prominent signals from metabolites (Figure S1). The global fecal metabolite composition was significantly different in HFD-C from mice fed either of the fermentable carbohydrates as determined by principal components analysis (PCA), whereby two clusters were observed in the scores plots relating to HFD-I and HFD-BG versus HFD-C (Figure S2). The variation in the first two principal components was dominated by consumption of fermentable carbohydrate, such that no time trajectory patterns were observed.

Statistics for various pairwise OPLS-DA comparisons between different fermentable carbohydrates are shown in Table S2. No significant difference was observed for the comparison between HFD-BG and HFD-I. However, clear differentiation of both HFD-I and HFD-BG from the HFD-C group was confirmed by orthogonal partial least squares discriminant analysis (OPLS-DA) which gave Q^2^ Yhat values of 94.1% and 85.6% respectively for HFD-I and HFD-BG at week 8 (Table S2), indicating that the separations between these models are highly robust.

**Figure 5:**
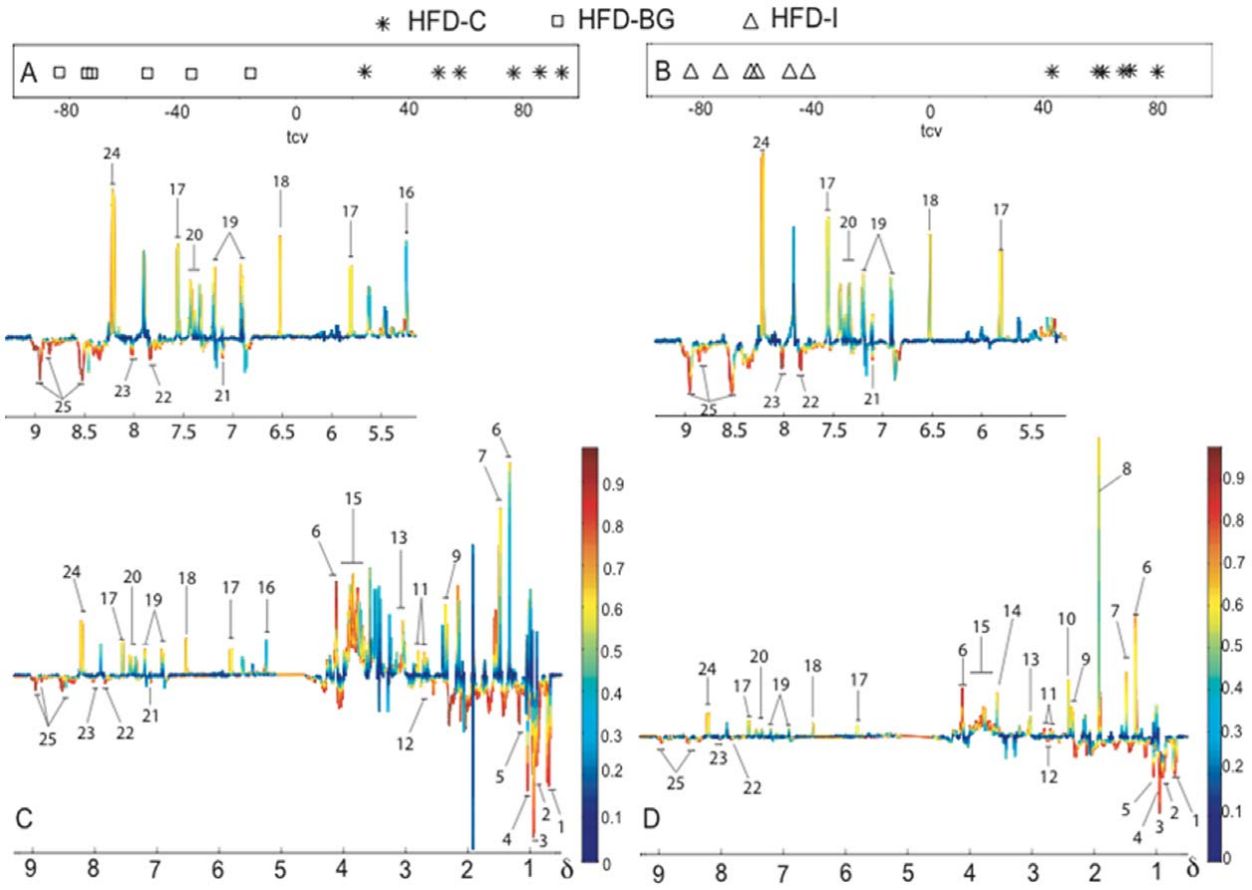
Multivariate statistical analysis of the fecal ^1^H NMR spectra. OPLS-DA cross validated scores plots for mice fed with (a) HFD-C and HDF-BG; and (b) HFD-C and HFD-I. The corresponding coefficient plots indicated fecal metabolic differences for (c) HFD-C and HFD-BG; and (d) HFD-C and HFD-I. Insets show an expansion of the aromatic region. HFD-C, high fat diet control; HFD-I, high fat diet+inulin; HFD-BG, high fat diet+β-glucan. 1, Bile acids; 2, Butyrate; 3, Isoleucine, leucine and valine; 4, Propionate (tentative); 5, Unknown at δ 1.17 (doublets) ; 6, Lactate; 7, Alanine; 8, Acetate; 9 Glutamate (tentative); 10, Succinate; 11, Aspartate; 12, Citrate; 13, Lysine; 14, Glycine; 15, Glucose and amino acids; 16, Glucose; 17, Uracil; 18, Fumarate; 19, Thyrosine; 20, Phenylalanine; 21, Histidine; 22, Unknown at δ7.84 (doublets); 23, Unknown at δ8.02 (doublets); 24, Unknown at δ8.20 (doublets); 25, Amine related compounds.

The regression coefficient plots indicated that lactate; citric acid cycle intermediates (succinate, fumarate); amino acids (lysine, alanine, glutamate, aspartate, and glycine); and aromatic compounds (uracil, tyrosine and phenylalanine) were the dominant metabolites in the feces of HFD-I and HFD-BG groups. Fecal glucose level was characteristic of HFD-BG mice at week 8. The integration of ^1^CH glucose signal at δ5.230–5.262 showed that glucose was approximately three times greater in the feces from HFD-BG mice compared to HFD-I and HFD-C groups. Bile acids; possibly amine related compounds (characterized by the broad singlets in δ8.5, 8.85 and 8.95); and an unknown metabolite at δ1.17 were highly correlated to HFD-C mice (shown for week 8 in Fig. 5).

## Discussion

In the present study, we confirm the finding of others that both β-glucan [30] and inulin [7], [31] attenuate weight gain in high fat fed mice. Although both groups that were fed fermentable carbohydrates displayed a similar significant decrease in cumulative body weight gain compared to the high fat control group, the significant decline in cumulative food intake occurred earlier (HFD-BG, week 4 vs. HFD-I, week 6), and was significantly greater in the HFD-BG compared with HFD-I group. However, a significantly greater reduction in body adiposity was observed in the HFD-I group as compared to HFD-BG and HFD-C groups. Considering both the HFD-BG and HFD-I have the similar end body weight reduction, it would suggest that energy intake alone does not account for weight loss and change in body composition in the HFD-I group. Consistent with the decrease in body weight HFD-BG and HFD-I had lower body fat that HFD-C with HFD-I significantly lower than the other two groups. Although not significantly different between HFD-BG and HFD-I, a trend towards a lower internal fat than HFD-C was observed, with HFD-I having a smaller epididymal fat pad. It is possible that inulin may have a preferential effect on adipose tissue. The mechanism by which inulin displays a greater effect on adipose tissue is unclear, although we and others have reported impact of inulin on body fat content previously [19]. There is evidence that inulin may affect fatty acid oxidation [32].

A ^1^H NMR spectroscopy-based metabolomic approach was used to characterize the metabolic profiles of the feces. Here, no significant difference was observed between the ^1^H NMR spectra of the HFD-I and HFD-BG groups. However, compared to HFD-C, the effects of the β-glucan and inulin on body weight reduction were coupled with the increased excretion of amino acids, citric acid cycle intermediates and lactate. This suggests enhanced fecal energy loss, in both groups through these energy related compounds. It also reflects reduced utilization of these energy related compounds that may have led to reduced body weight gain in fermentable carbohydrates fed groups under high fat dietary conditions. The HFD-BG group had increased fecal excretion of glucose at week 8, which may indicate incomplete fermentation of β-glucan. This may be an indication of an additional loss of energy in the HFD-BG.

Next we investigated if the differences in energy intake between the HFD-BG and HFD-I could be ascribed to changes in the gut microbiota. The high fat control diet had little effect on fecal total microbiota temporally, as measured by FISH at repeated intervals, over the 8 weeks dietary intervention. However, supplementation of both fermentable carbohydrates resulted in modulation of gut microbiota leading to an increase in total *Lactobacillus-Enterococcus* and *Bifidobacterium* both in feces and cecal contents over the 8 weeks dietary intervention. This suggests similar prebiotic efficacy of both fermentable carbohydrates in modulating the microbiota and supports previous reports on the prebiotic potential of oligofructose enriched inulin [4] and β-glucan [5]. Our results also support previous observations that increased intestinal bifidobacteria relates to reduced body weight gain and food intake [33]. Thus, there is the potential for utilizing fermentable carbohydrates to modulate the gut microbiota to ameliorate the detrimental effects induced by high fat diets, which is supported by our current data, but this does not fully account for the difference in energy intake.

Recent evidence has implicated the ratio of firmicutes:bacteroidetes as one of the environmental factors regulating energy homeostasis. It appears that obese mice and humans possess more bacterial groups belonging to firmicutes, which possess fermentation ability to extract energy from otherwise indigestible carbohydrates [2]. The energy derived from these indigestible carbohydrates in the form of SCFAs contributes to total energy in adults [34] and to the obese phenotype. However, in the present study, we observed higher proportions of lactobacilli belonging to firmicutes as well as enhanced cecal bacteroides number, (as evident from increased cecal MIB counts) coupled with higher total colonic SCFA content in HFD-I and HFD-BG compared with HFD-C, accompanied by a reduction in body weight. Using high throughput analysis, Everard *et al* also demonstrated increase in bifidobacteria and bacteroidetes with oligofructose supplementation in ob/ob mice. However, they reported decrease in firmicutes coupled with modulation of actinobacteria and proteobacteria populations [35]. Our previous work has demonstrated that fermentable carbohydrates can effect hypothalamic neuronal activation [19]. MEMRI is an imaging technique which gives a measure of neuronal activation *in vivo*. Manganese ions (Mn^2+^) enter into the excitable neuronal cells in the brain producing contrast in the MRI images. The appetite centers are located in the hypothalamus, that include the arcuate nucleus which is comprised of neurons expressing orexigenic (neuropeptide Y, agouti related peptide) and anorexigenic (proopiomelanocortin, melanocyte stimulating hormone) peptides, which project into other areas like VMH and PVN. Brainstem is another region which acts as the receiver of vagal afferent signals from all along the gastro intestinal tract and all the signals are transmitted to hypothalamus [19], [20]. We demonstrated a significant decrease in the signal intensity due to manganese uptake, measured in the ARC, VMH, PVN in the HFD-BG group. These patterns of activation are similar to those previously reported with acute infusion of anorectic gut hormones such as PYY and GLP-1 [36], [37].The results, therefore, reflect a satiated state in β-glucan fed mice, which is paralleled by the significantly lower and earlier decline in the cumulative food intake. A similar decrease in VMH and PVN nuclei has earlier been reported in resistant starch fed mice [11]. It has been shown that a decrease in these basal levels of neural activity is an effect of a satiated state, in a study comparing fed and fasted neural activity in these hypothalamic nuclei [26]. Therefore, despite the fact that there are populations of neurons present in the arcuate nucleus, for example, which have opposing effects on appetite, the overall effect detected with MEMRI links a higher activation with a fasted state [26].

We expected a similar reduction in the signal intensity of HFD-I group, but instead MEMRI patterns resembled those of the HFD-C, except for in the NTS. NTS is located in brainstem, the significantly lower MEMRI signal in NTS in inulin fed animals was not coupled with parallel lower signal intensity in the hypothalamic appetite regions. This is a surprising finding, raising the possibility of different mechanisms of action behind the anti-obesity potential of these two different fermentable carbohydrates. The peripheral changes produced due to inulin supplementation in diet were reflected in brainstem but it did not produce satiated state in mice as compared to HFD-BG. A further explanation may lie in the differences in viscosity and solubility of two different fermentable carbohydrates. Whereas inulin is highly soluble, beta-glucan forms a viscous solution in water. It has been reported that viscosity better explains reduction in energy intake as compared to solubility and fermentability of fibers [38].

Increased expression of the anorectic gut hormones GLP-1 and PYY mRNA in the colon and increased portal GLP-1 concentrations in fermentable carbohydrate fed rodents have been reported previously [7], [39], [40]. The effect was attributed to the increased level of SCFAs in these studies. However, despite higher concentration of SCFA in the feces of both the inulin and β-glucan groups, we found no significant differences for PYY levels as well as colonic expression between the groups, due to limited tissue and plasma we were unable to measure GLP-1. We suggest that our observations link with a potential role of SCFA impacting on peripheral metabolism rather than colonic anorectic gut hormones.

SCFA have been shown to have direct metabolic effects on adipocytes through the SCFA receptor FFAR2 (Free Fatty Acid Receptor 2) causing a reduction of FFA output in adipocytes [41]. The SCFA receptor FFAR3 (Free Fatty Acid Receptor 3) has been shown to be expressed in sympathetic nervous system ganglia [42] and its stimulation increases energy expenditure. In the present study, total SCFA content was highest in HFD-BG followed by HFD-I as compared to HFD-C. Therefore, it is possible to speculate that different levels and patterns of SCFAs produced may cause differential activation of FFAR2 and FFAR3 in adipose tissue and appetite centers in brain, respectively, leading to the different effects of inulin and β-glucan observed in the present study.

In conclusion, this study supports the work of others and demonstrates that dietary supplementation of inulin or β-glucan limits weight gain in high-fat fed animals, however, it highlights the multiple mechanisms involved to explain these observations. β-glucan supplementation appears to have a greater impact on energy intake than inulin despite both groups ending at the similar weight reduction. Supplementation of both compounds caused energy loss in the feces, a positive change in the microbiota, with no change in colonic PYY content. Inulin appears to have a greater effect on limiting total adipose tissue content and adipocyte size, whereas β-glucan affected appetite regulation by suppressing neuronal activation in the hypothalamic nuclei implicated in the appetite high fat control. The study highlighted the multi-factorial nature of these molecules on energy homeostasis. Understanding of the different mechanisms involved may open up new strategies for nutritionally targeting obesity.

## Supporting Information

Method S1 **Supplementary information for detailed methods used.**
(DOCX)

Table S1 **Composition of diets.**
(DOCX)

Table S2 **Pair wise OPLS-DA models assessed by Q^2^Yhat and the significance of model based on 500 times random permutation of Y matrix.**
(DOCX)

Figure S1 **Median fecal ^1^H NMR spectra of mice (a) HFD-C (b) HFD-BG and (c) HFD-I.**
(TIF)

Figure S2 **PCA scores plot of fecal metabolite profiles showing clear clustering patterns for HFD-C, HFD-BG and HFD-I groups of mice.**
(TIF)
