# Supplementary material for: Differential Effects of Two Fermentable Carbohydrates on Central Appetite Regulation and Body Composition
Source: PLoS One. 2012 Aug 29;7(8):e43263. doi: 10.1371/journal.pone.0043263 (PMC3430697; doi:10.1371/journal.pone.0043263)
Supplement: Method S1 — Supplementary information for detailed methods used. (DOCX) [file pone.0043263.s001.docx]

**Method S1. Supplementary information for detailed methods used.**

**Fluorescent in situ hybridization (FISH) to assess microbial changes :** The protocol was followed as previously described by Marin-Pelaez *et al.*, 2008 (12). Briefly, frozen fecal and cecal samples were diluted in 10 volumes of phosphate buffered saline PBS (pH 7.2; Oxoid). The samples were homogenized and supernatant fractions (375 μl) containing bacterial cells were fixed with 4% (w/v) paraformaldehyde in a ratio of 1:3 at 4 ^o^C. Bacterial cells were subjected to centrifugation (1000xg; 3 mins), washed twice with PBS and finally suspended in an equal volume of PBS and ethanol (150 μl each). Cells were appropriately diluted and 20 μl of the suspension was applied to teflon poly-L-lysine coated 6-well glass slides (Tekdon Inc., USA). Permeabilization of cells was carried out in an ethanol series (50, 80 and 96%) for 3 min each and hybridized with genus specific Cy-3 labeled (at 5’ end) probes for 4 hr at 50 ^o^C. The probes used were Lab158 (13), Bif164 (14), Erec482 (15)and Mib663 (16) to enumerate *Lactobacillus-Enterococcus*, bifidobacteria, *Eubacterium rectale-Clostridium coccoides* and mouse intestinal bacteria, respectively. The slides were subsequently washed, mounted in ProLong^®^ Gold antifade reagent and stored at 4 ^o^C under dark conditions. The fluorescent cells were manually counted in 15 random fields of view per well. Total cell counts were obtained with DAPI (4′, 6-diamidino-2-phenylindole dihydrochloride) staining.

**1H NMR spectroscopy for metabolic profiling of feces:** Fecal samples for 6 mice per group were prepared and analysed by ^1^H NMR using method adapted from Saric et al (16). Each stool pellet was homogenized with 1mL buffer and sonicated at 25°C for 30 minutes and then centrifuged at 20,000xg for 20 minutes. Supernatant (650 μL) were transferred into a new Eppendorf tube and stored at -40 °C. Prior to data acquisition, the fecal supernatant was defrosted, centrifuged at 20,000x g and 500 μL of the supernatant transferred into NMR tubes of 5 mm outer diameter. A one-dimensional (1D) ^1^H NMR spectra of fecal water samples were acquired for each sample using a Bruker DRX 600 NMR spectrometer operating at 600.29 MHz for ^1^H equipped with TXI probe (Bruker; Rheinstetten, Germany) using pulse sequence with water suppression using excitation sculpting. For each sample, 256 scans were collected into 64K data points over a spectral width of 20 ppm with a relaxation delay of 4 s and an acquisition time of 2.73 s. The spectra were resolution-enhanced through use of the exponential window function (with LB = 1) prior to Fourier Transformation.

**Gas chromatography for determination of colonic content of short chain fatty acids:** Short chain fatty acids (SCFA) in the colonic content were determined by gas chromatography analysis. Cecal contents were weighed (20-220 μg) and combined with 550 μl of PBS. Samples were vortex mixed for 1 minute, centrifuged at 3000x g for 10 minutes and the supernatant collected. To 500 μl supernatant, 25 μl of internal standard, 2-ethylbutyric acid, was added to give a final concentration of 5 mmol/l. Acids were extracted by the addition of 250 μl concentrated hydrochloric acid and 1 ml diethyl ether followed by vortex mixing for 1 minute. Samples were centrifuged for 10 minutes at 3000xg and the ether layer removed and transferred to a separate capped vial. *N*-methyl-*N*-t-butyldimethylsilyltrifluoroacetamide (MTBSTFA; Sigma) was added (100 μl) before heating at 80°C for 20 minutes.

Gas chromatography was performed on a Hewlett Packard 5890 Series II instrument equipped with a flame ionization detector, split/splitless injector and a 10 m, 0.18 mm ID and 0.20 μm df Rtx^®^-1 (Crossbond^®^ 100% dimethyl polysiloxane, Thames Restek UK, Ltd) capillary column. Injector and detector temperatures were 275°C with the column temperature programmed from 63°C for 3 minutes to 190°C at 10 °C/minute. Helium served as the carrier gas (head pressure 135 kPa) and injections (1 μl) were made in the split mode (50:1 split). Peak areas were recorded and all subsequent data manipulation was completed using ChemStation Software (Agilent Technologies). External standards for acetate, propionate, *n*-butyrate, iso-butyrate, *n*-valerate and caproate were prepared at concentrations of 25, 12.5, 6.25, 1.25 and 0.625 mM and ethyl butyric acid was used as the internal standard at a concentration of 100 mM. Reported values were normalized according to the weight of original sample used.
