# Supplementary material for: Differential Effects of Two Fermentable Carbohydrates on Central Appetite Regulation and Body Composition
Source: PLoS One. 2012 Aug 29;7(8):e43263. doi: 10.1371/journal.pone.0043263 (PMC3430697; doi:10.1371/journal.pone.0043263)
Supplement: Table S1 — Composition of diets. (DOCX) [file pone.0043263.s002.docx]

| **Diets** | **HFD-C** | **HFD-I** | **HFD-BG** |
| --- | --- | --- | --- |
| Ingredients | g/kg | g/kg | g/kg |
| Energy (Kcal/g) | 4.6 | 4.6 | 4.6 |
| Casein | 195.0 | 195.0 | 195.0 |
| DL- Methionine | 3.0 | 3.0 | 3.0 |
| Sucrose | 342.96 | 342.96 | 342.96 |
| Corn Starch | 75.0 | 75.0 | 75.0 |
| Maltodextrin | 75.0 | 75.0 | 75.0 |
| Anhydrous milk fat | 210.0 | 210.0 | 210.0 |
| Cellulose | 110.0 | 50.0 | 50.0 |
| Mineral Mix (AIN-76) | 35.0 | 35.0 | 35.0 |
| Calcium Carbonate | 4.0 | 4.0 | 4.0 |
| Vitamin Mix (Teklad) | 10.0 | 10.0 | 10.0 |
| Ethoxyquin, antioxidant | 0.04 | 0.04 | 0.04 |
| Inulin | 0.0 | 100.0 | 0.0 |
| β-glucan | 0.0 | 0.0 | 100.0 |
