# Supplementary material for: Differential Effects of Two Fermentable Carbohydrates on Central Appetite Regulation and Body Composition
Source: PLoS One. 2012 Aug 29;7(8):e43263. doi: 10.1371/journal.pone.0043263 (PMC3430697; doi:10.1371/journal.pone.0043263)
Supplement: Table S2 — Pair wise OPLS-DA models assessed by Q2Yhat and the significance of model based on 500 times random permutation of Y matrix. (DOCX) [file pone.0043263.s003.docx]

| **Pairwise**  **OPLD-DA models** | **Data on week** | **Q^2^Yhat**  **(%)** | **Model**  **P-value** |
| --- | --- | --- | --- |
| HFD-C vs HFD-BG | 4 | 85.0 | 0.002 |
|  | 8 | 85.6 | 0.002 |
|  |  |  |  |
| HFD-C vs HFD-I | 4 | 90.7 | 0.002 |
|  | 8 | 94.1 | 0.002 |
|  |  |  |  |
| HFD-I vs HFD-BG | 4 | 47.1 | 0.03 |
|  | 8 | n/a | n/a |
