# Supplementary figures and images for: Differential Effects of Two Fermentable Carbohydrates on Central Appetite Regulation and Body Composition
Source: PLoS One. 2012 Aug 29;7(8):e43263. doi: 10.1371/journal.pone.0043263 (PMC3430697; doi:10.1371/journal.pone.0043263)

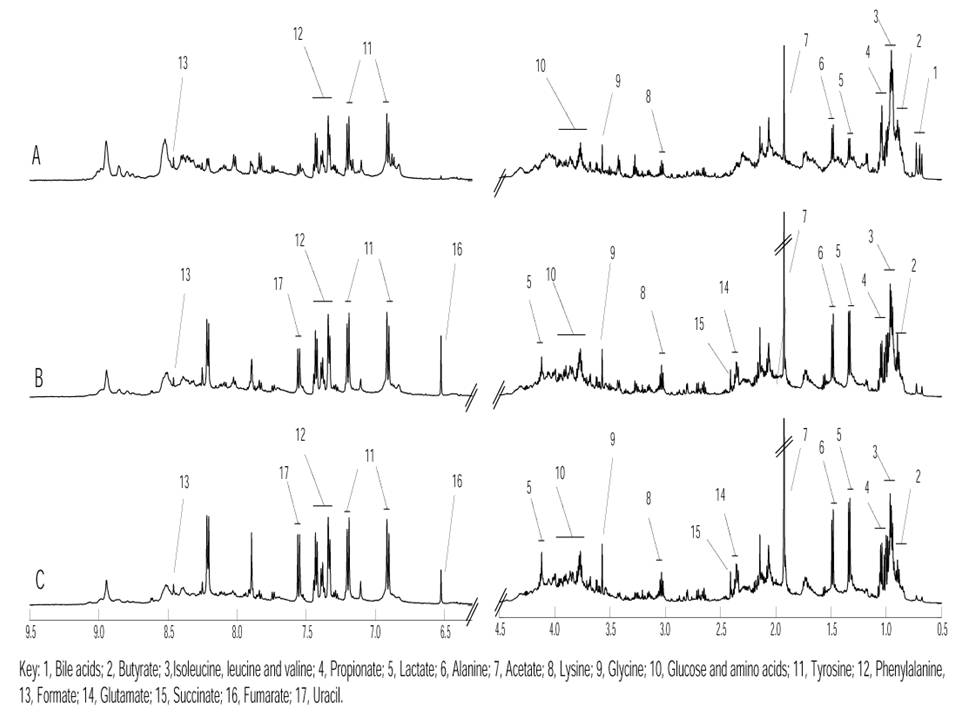

Supplement: Figure S1 — Median fecal 1H NMR spectra of mice (a) HFD-C (b) HFD-BG and (c) HFD-I. (TIF) [file pone.0043263.s004.tif]

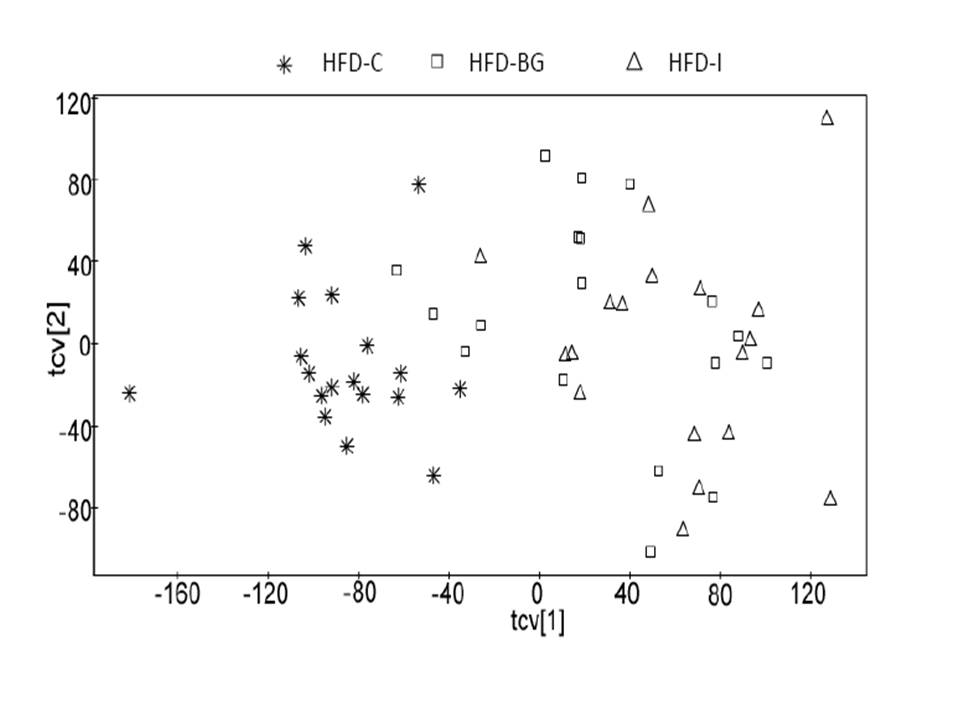

Supplement: Figure S2 — PCA scores plot of fecal metabolite profiles showing clear clustering patterns for HFD-C, HFD-BG and HFD-I groups of mice. (TIF) [file pone.0043263.s005.tif]
